# High species diversity and biochar can mitigate drought effects in arid environments

**DOI:** 10.3389/fpls.2025.1563585

**Published:** 2025-05-20

**Authors:** Hamada E. Ali, Ahmed M. Al-Wahaibi

**Affiliations:** ^1^ Department of Biology, College of Science, Sultan Qaboos University, Al-Khoud 123, Muscat, Oman; ^2^ Life Science Unit, College of Science, Sultan Qaboos University, Al-Khoud 123, Muscat, Oman

**Keywords:** arid lands, drought, plant species diversity, microbial content, oman

## Abstract

**Introduction:**

Climate change, including drought, threatens ecosystems across the globe. The current study investigated the effects of species diversity and biochar application on the performance and productivity of five native Omani species under control and drought conditions.

**Methods:**

A fully controlled greenhouse experiment was conducted in which five native species of three different diversities (one, two, and four species) were grown under four different treatments: biochar+drought, biochar, drought, and control. Productivity was measured through total biomass and root-to-shoot ratio), while performance was assessed in the form of plant functional traits (plant height, specific leaf area (SLA), and specific root length (SRL). Nutrient availability in the soil was measured using soil organic carbon (SOC) and soil total nitrogen (STN). Soil microbial content was determined using soil microbial biomass “Cmic” and soil microbial basal respiration. Biodiversity effects were analyzed using the complementarity effect (CE), selection effect (SE), and net biodiversity effect (NBE).

**Results and discussion:**

The study indicated that high diversity and biochar resulted in: 1. species with 66.6% greater total biomass and a 3% reduction in RSR, 2. enhanced species performance, with plants growing 25% taller, 50% higher SLA, and 25% higher SRL, 3. more fertile soil, with SOC and STN increasing by 40% and 33.3%, respectively, and 4. improved microbial content, with Cmic rising by 15% and basal respiration increasing by one-third under drought conditions compared to monoculture. These results highlight the intricate interactions between climate change and biodiversity, which are crucial for predicting the impact of changes in functional composition on ecosystem processes and, subsequently, for restoring arid ecosystems in Oman.

## 1 Introduction

Arid and semi-arid ecosystems comprise approximately 40% of the Earth’s surface (Tariq et al., 2024) and are significantly influenced by climate change, particularly through drought, changes in land-use, and various human activities (Sala et al., 2000; Jung et al., 2020). Drought has complex effects on these ecosystems, impacting community composition and species diversity, and consequently, the functions and productivity of the ecosystem (Jung et al., 2020; Wei et al., 2022).

One of the important driver of community composition, ecosystem function, and climate change mitigation is the net biodiversity effect (Xu et al., 2024), which is mediated by complementarity and selection effects (Hu et al., 2021). The net biodiversity effect refers to the overall impact of species diversity on ecosystem functioning, particularly primary productivity (Chen et al., 2025). Studies have consistently shown a positive relationship between biodiversity and ecosystem productivity across various ecosystems (Bruno et al., 2005; Maron et al., 2021). Complementarity effects occur when species in diverse communities use resources more efficiently through niche differentiation or facilitation, leading to increased ecosystem productivity and stability (Wang et al., 2021). Selection effects occur when dominant species with particular traits affect ecosystem processes, which can be positive, negative, or neutral (Chen et al., 2025). A positive selection effect occurs when species with particular traits dominate communities and enhance ecosystem processes (Loreau and Hector, 2001), whereas a negative selection effect occurs when no dominant or high-performing species are found within the community (Jiang et al., 2008). Several studies have reported that plant diversity promotes ecosystem functioning through biomass production (Huang et al., 2020; Hu et al., 2021; Xi et al., 2023), however a robust conclusion regarding such role is still missing. Studies have shown that the biodiversity effects “net biodiversity, complementarity and selection effects” are mediated by the plant functional traits (e.g., plant height, specific leaf area (SLA), specific root length (SRL), and root to shoot ratio (RSR)) of the plant species that make these communities (Roscher et al., 2012). Plant height measures plant competitiveness in capturing resources, such as light (Pérez-Harguindeguy et al., 2013), SLA reflect growth rates (Violle et al., 2012), whereas SRL show the economic aspects of root systems as it measures the length to mass ration of the root system (Ostonen et al., 2007), and RSR which show the ability of plants to mitigate drought conditions, especially in arid ecosystem (Cambui et al., 2011).

In addition to plant diversity and functional traits, soil properties and microbial content are important for determining ecosystem functionality and resilience (Martinez-Almoyna et al., 2020). Soil organic carbon (SOC) and soil total nitrogen (STN) are important indicators of soil fertility, which are affected by various biotic and abiotic factors (Crowther et al., 2016; Gibbons et al., 2017). Additionally, SOC and STN affect multiple functional processes in the soil, including its ability to retain water and maintain stable aggregates (Murphy, 2015). On the other hand, soil microbial communities (microbial carbon “Cmic” and soil basal respiration) considered as important drivers of ecosystem functions, particularly for nutrient cycling and decomposition processes, especially in arid ecosystems (Wang et al., 2020b).

It has been shown that higher plant diversity increases soil microbial biomass, respiration, and fungal abundance, which consequently improve nitrogen mineralization rates (Schnitzer et al., 2011). This relationship between plant diversity and soil microbial content is complex, as plant diversity influences soil microbial community structure and function, while soil microbes reciprocally affect plant performance and productivity, and in turn, ecosystem processes (Saleem et al., 2019; Jayaramaiah et al., 2025). Several previous studies have reported a relationship between plant diversity and soil microbial properties, which is not always straightforward. For instance, in temperate deserts, soil bacterial alpha and beta diversities are primarily determined by abiotic and spatial factors, followed by plant factors (Wang et al., 2020b). Additionally, the temporal effects of plant diversity on soil microbial content can change over time, suggesting that the recovery of soil microbial communities from land-use changes may take more than a decade (Strecker et al., 2016). Such properties can be significantly enhanced by soil management practices, such as biochar amendments, in areas that suffer from low diversity and drought, especially arid and semi-arid ecosystems (Ali et al., 2023). Biochar amendment can improve soil properties and soil microbial activities, which can consequently improve ecosystem structure and functions (Joseph and Lehmann, 2015; Mandal et al., 2016). It is considered an eco-friendly method to mitigate the effects of climate change (Griscom et al., 2017), especially adapting soil to extreme weather events, such as prolonged drought (Chausson et al., 2020) and as a result, benefits biodiversity (Morecroft et al., 2019; Seddon et al., 2021). Biochar is a carbon-rich material produced by biomass pyrolysis in an oxygen-limited environment (Lehmann et al., 2015). It enhances soil fertility directly by providing essential soil nutrients and soil carbon (Coomes and Miltner, 2017; Igalavithana et al., 2017) or indirectly by neutralizing soil acidity (Zhang et al., 2017) and increasing water holding capacity and soil aeration.

In the current study, a fully controlled greenhouse experiment was conducted to study the interactive effects of plant species diversity and biochar amendment, on plant performance, plant productivity, soil properties, soil microbial content and biodiversity effects under drought conditions using five native species to Oman (*Ranunculus muricatus* L., *Ipomoea obscura* (L.) Ker Gawl., *Pulicaria glutinosa* (Boiss.) Jaub. & Spach, *Dicoma schimperi* (DC.) Baill. ex O.Hoffm., *Paronychia arabica* (L.) DC.). Artificial plant communities were established using these five species with three levels of diversity (one, two, and four species). These communities were subjected to four treatments: control, drought, biochar, and biochar+drought. Specifically, the following questions were addressed:

1. Do species diversity and biochar amendment affect plant performance and productivity under drought conditions? How are these effects mediated by plant functional traits (plant height, SLA, and SRL)?
2. Does the relative importance of net biodiversity, complementarity, and selection effects change with species diversity and biochar amendment under drought conditions?
3. Are these effects controlled by soil properties (SOC and STN) and microbial content (Cmic and basal respiration)?

By integrating biodiversity, plant functional traits, soil properties, soil microbial content, and the use of biochar as an eco-friendly soil amendment, this study provides solutions for mitigating drought impacts in arid ecosystems that suffer from low species diversity. These findings shed light on the mechanisms driving ecosystem resilience, with practical implications for the restoration and conservation of arid environments under drought conditions.

## 2 Materials and methods

### 2.1 Plant species and experimental setup

**Figure 1:**
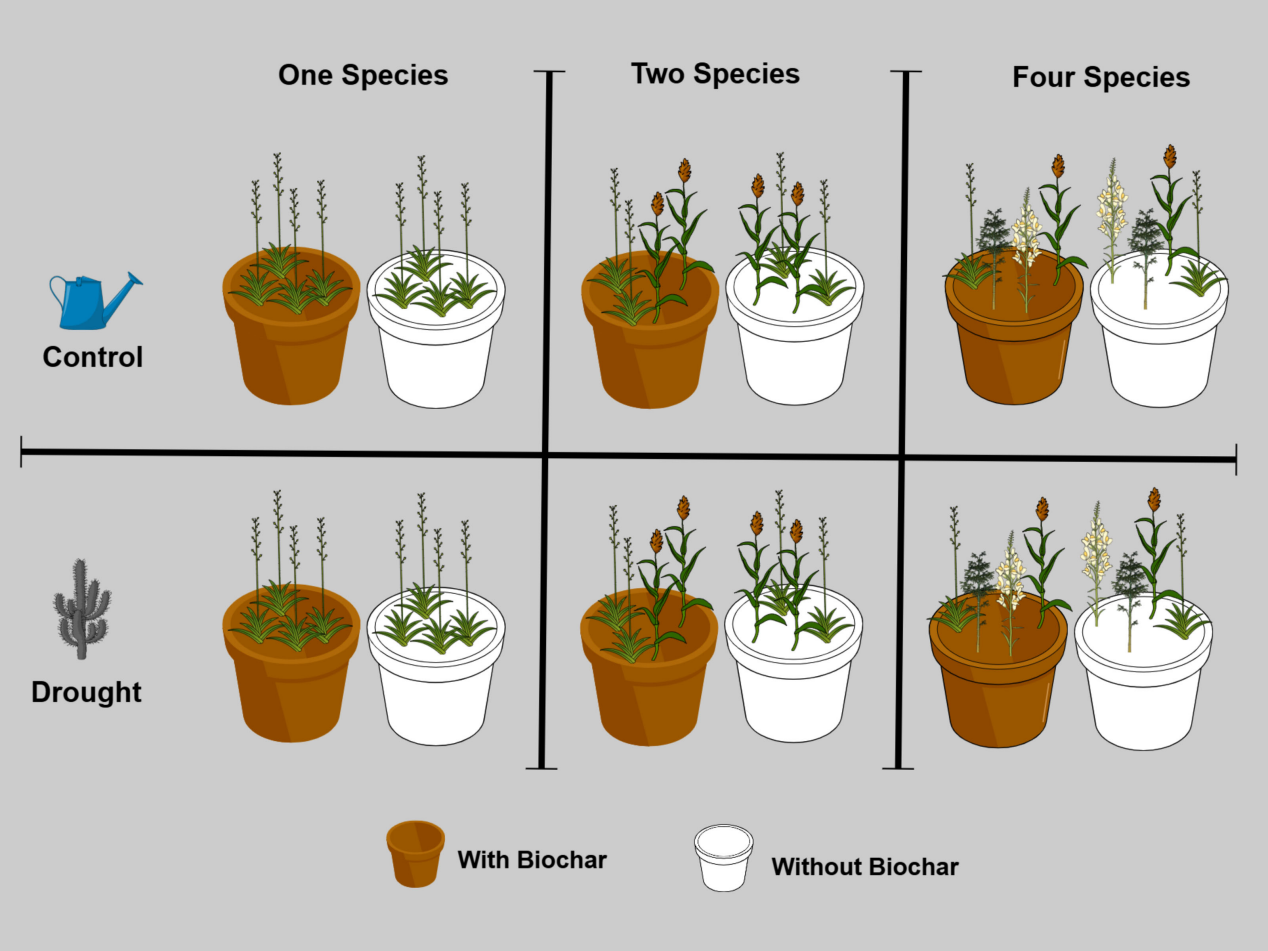
Experimental design to investigate the effects of drought (drought and control), biochar (with and without biochar), and species diversity (one, two, and four species), with five replicates per treatment (*n* = 60).

A full factorial experiment was conducted, including four treatments (Control, Drought, Biochar, and Biochar + Drought) with three levels of species diversity (one, two, and four species) and five replicates per treatment in the greenhouse of Sultan Qaboos University, Muscat, Oman (23 36’0.95″ N, 58°10’5.29″ E) (**Figure 1**). Five native species were chosen to construct the experimental plant communities, *Ranunculus muricatus* L., *Ipomoea obscura* (L.) Ker Gawl., *Pulicaria glutinosa* (Boiss.) Jaub. & Spach, *Dicoma schimperi* (DC.) Baill. ex O.Hoffm., *Paronychia arabica* (L.) DC. (**Supplementary Table S1** in the **Supplementary Materials**) based on their occurrence in the natural habitats of Oman (Pickering and Patzelt, 2008; Ghazanfar, 2015). For the treatments of the different species diversities, five monocultures (i.e., one for each species) were established for the 1-species treatment and five species mixtures for both the 2-species and 4-species treatments. Species mixtures were randomly selected from the original species pool, which consisted of five species, all of which had equal representation at each level of diversity (**Supplementary Table S2** in the **Supplementary Materials**). Each monoculture or species mixture was considered as one replicate of the diversity level, which resulted in five replicates for each of the three species diversity levels. In total the experiment had 60 pots (3 species diversity (1, 2 and 4 species) × 4 treatments “Control, Drought, Biochar, and Biochar + Drought” × 5 replicates).

On February 3^rd^, 2024, seeds of the five species were sown in separate trays (60 cm x 30 cm x 3 cm) filled with universal potting soil (Potgrond, Van Egmond Potgrond B.v, Rijnsburg, Holland) in the greenhouse under temperature controlled at 25°C during the daytime and 16°C during nighttime and watered twice a week. On March 5^th^, 2024, four seedlings of different species mixtures of the same size were transplanted into 60 pots (length = 50 cm, width = 21.5 cm, and height = 16 cm) (**Figure 1**; **Supplementary Tables S1, S2**) filled with pre-sieved soil collected by digging from the Sultan Qaboos University Botanic Garden. For the biochar treatment, 30 pots were amended with 1.25 kg of biochar to the topsoil before seedling transplantation; the other 30 pots did not receive any biochar, following the recommendation of Ali et al. (2023). All pots were randomly placed in the greenhouse and watered twice a week for three weeks, after which dead seedlings and seedlings of other species growing within the study pots were continuously removed throughout the experiment. To emulate the drought treatments, the pots were split into two treatments: the control pots (*n* = 30) which were watered normally with 660 ml twice a week, and the drought pots (*n* = 30) that were watered twice a week with just 220 ml, which represents 20% of soil saturation following previous studies (Ali and Bucher, 2022; Ali et al., 2023).

### 2.2 Plant harvest and measurements

On September 10, 2024, aboveground and belowground plant functional traits (plant height, SLA, SRL, and RSR) were measured along with total biomass following standardized protocols (Pérez-Harguindeguy et al., 2013). To account for intraspecific trait variability, traits were measured for every individual within each pot (Albert et al., 2012; Ali et al., 2017a). Plant height (cm) was measured as the distance between the lowest and highest photosynthetic parts of the plant using a meter. On three healthy and fully developed leaves for every individual in each pot, SLA was measured as the ratio between leaf area (LA, mm^2^) and dry mass (mg), expressed as (mm^2^ mg^-1^). LA was measured digitally by analyzing scanned digital images using Easy Leaf Area software (Easlon and Bloom, 2014). The leaves were weighed to record the fresh mass, oven-dried at 70°C for 48 h, and then weighed again to assess the leaf dry mass (mg). Finally, LA was divided by the leaf dry weight to calculate SLA. The biomass and root-to-shoot ratio (RSR) of the plants were calculated after cutting the plants at the soil surface, drying them at 70°C for 48 h, and weighing them as aboveground biomass (g). Additionally, the RSR for each individual was measured as the ratio of root dry weight to shoot dry weight, as described by Mašková and Herben (2018). Finally, the SRL (cm/g) was measured as the root length divided by the root dry mass after scanning the whole root using a flat-bed scanner at a resolution of 800 dpi, and measured root length (cm) for each individual using the images scanned by RhizoVision Explorer (Seethepalli et al., 2021).

After harvesting the aboveground and belowground plant materials from all pots, the soil was mixed within each treatment and air-dried to measure the soil organic carbon (SOC) and soil total nitrogen (STN), which reflect soil fertility, soil quality, and consequently plant growth (Crowther et al., 2016). SOC was determined using a modified Walkley and Black wet oxidation method (Nelson et al., 1996) and STN was determined using the modified macro Kjeldahl digestion method (Bremner and Mulvaney, 1983); both were expressed as g/kg soil.

To understand how drought, biochar, and species diversity may affect plant performance and production by affecting soil microbial activity, soil microbial biomass and soil microbial basal respiration were measured using 3 g of fresh soil with an O_2_-microcompensation apparatus following Scheu (1992). Soil microbial basal respiration (μl O_2_ h^-1^ g^-1^ dry soil) was measured hourly for 24 h at 20°C. Additionally, soil microbial biomass (Cmic; μg C g^-1^ dry soil) was calculated from the maximum initial respiratory response after the addition of glucose, as described by Eisenhauer et al. (2007).

Finally, the complementarity effect (CE), selection effect (SE), and net biodiversity effect (NBE) were measured to understand the effect of biodiversity under different diversity levels and treatments, following Loreau and Hector (2001). NBE was measured as the difference between the observed biomass of the species when grown in a mixed community and the expected biomass of the species in monoculture, based on the relative abundance of the species in the mixture, using the following equations:


NBE = Observed Biomass − Expected Biomass

$$Expected\;Biomass=\;\sum_{i=1}^{N}p_{i\;}\;x\;M_{i}$$

Where *N* is the number of species in the mixture, pi is the relative abundance of species i in the mixture, and Mi is the biomass of species i in monoculture. CE was measured using the following equation:


$$\text{CE}\;=\;N\;x\;\bar{\Delta \;RY_{i}}\;x\;\bar{M_{i}}$$

Where *N* is the number of species in the mixture, $\bar{\Delta \;RY_{i}}$ is the mean deviation of the relative yield of species *i* from its expected relative yield ( $RY_{i}-\;\frac{1}{N}$ ), and $\bar{M_{i}}$ is the mean monoculture yield of species *i*. Finally, the SE was calculated as the following formula as covariance between the relative yield ( $RY_{i}$ ) of species *i* and its monoculture yield ( $M_{i}$ ) multiplied by the number of species in the mixture (s).


$$\text{SE}\;=\;N\;x\;\text{cov}\left(RY_{i},\;M_{i}\right)$$

### 2.3 Statistical analysis

R software version 4.4.3 were used to run all statistical analyses and produce all figures (R Development Core Team, 2025). The effects of diversity (i.e., 1, 2, and 4 species) and the four combinations of biochar and drought treatments (control, drought, Biochar and Biochar + drought) were tested as fixed variables alone and in interaction on community productivity (i.e., biomass and RSR), plant functional traits (plant height, SLA, and SRL), soil abiotic properties (i.e., soil organic carbon (SOC) and soil total nitrogen (STN) contents), soil microbial activity (i.e., microbial biomass (Cmic) and basal respiration), and biodiversity effects (CE, SE, and NBE) as response variables. In addition, the plot ID was used as a random intercept using linear mixed effect models of the package “*lme4*” (Bates et al., 2015). For *post hoc* evaluation, Tukey’s multiple comparison test was applied between all the levels of the two fixed variables using the “*emmeans*” and “*multcomp*” packages (Hothorn et al., 2008; Lenth, 2022).

## 3 Results

Overall, the results of the current study showed significant effects of species diversity (two and four species), biochar and drought treatments (drought, Biochar and Biochar + drought), and their interactions on plant performance (plant height, SLA, and SRL), productivity (biomass and RSR), soil properties (SOC and STN), microbial content (Cmic and basal respiration), and biodiversity (NBE and CE) (**Supplementary Table S3**).

### 3.1 Effects of species diversity, biochar, and drought on species performance

**Figure 2:**
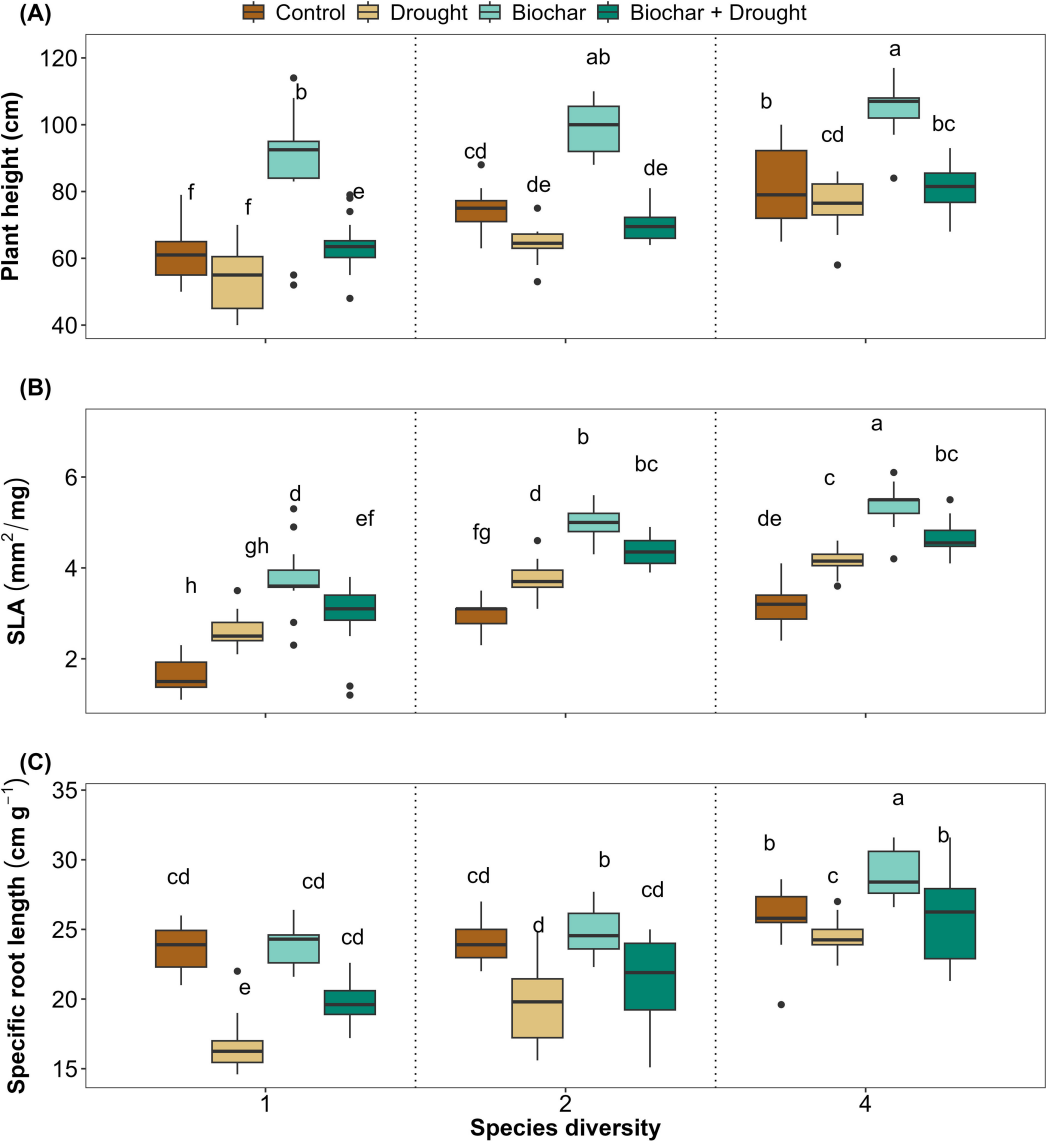
Effect of diversity and biochar application on **(A)** Plant height, **(B)** Specific leaf area (SLA), and **(C)** Specific root length (SRL) under control and drought conditions. Letters indicate significant differences among treatments based on Tukey’s multiple comparison test.

Significant differences were found in the three plant functional traits that reflected plant performance (plant height, SLA, and SRL) in response to diversity, biochar, and drought (**Figures 2A-C**). Biochar significantly improved plant height and SLA compared to the control treatments, even under drought conditions. Such improvements were positively correlated with species diversity for plant height, SLA, and SRL, and significantly increased with increasing species diversity. Although drought negatively affected SRL, 4-species diversity had a significant positive effect on SRL. Biochar alone did not have any significant effect on SRL, but it improved significantly under drought conditions and when interacting with 2- and 4-species diversities. These results were confirmed by the LMMs, which explained 76%, 86%, and 71% of the variance by plant height, SLA, and SRL, respectively (**Supplementary Table S3** in the **Supplementary Materials**). Although the interaction between species diversity, biochar, and drought did not significantly affect plant height and SLA, it significantly affected SRL (**Figures 2A-C**; **Supplementary Table S3**).

### 3.2 Effects of species diversity, biochar and drought on species productivity

**Figure 3:**
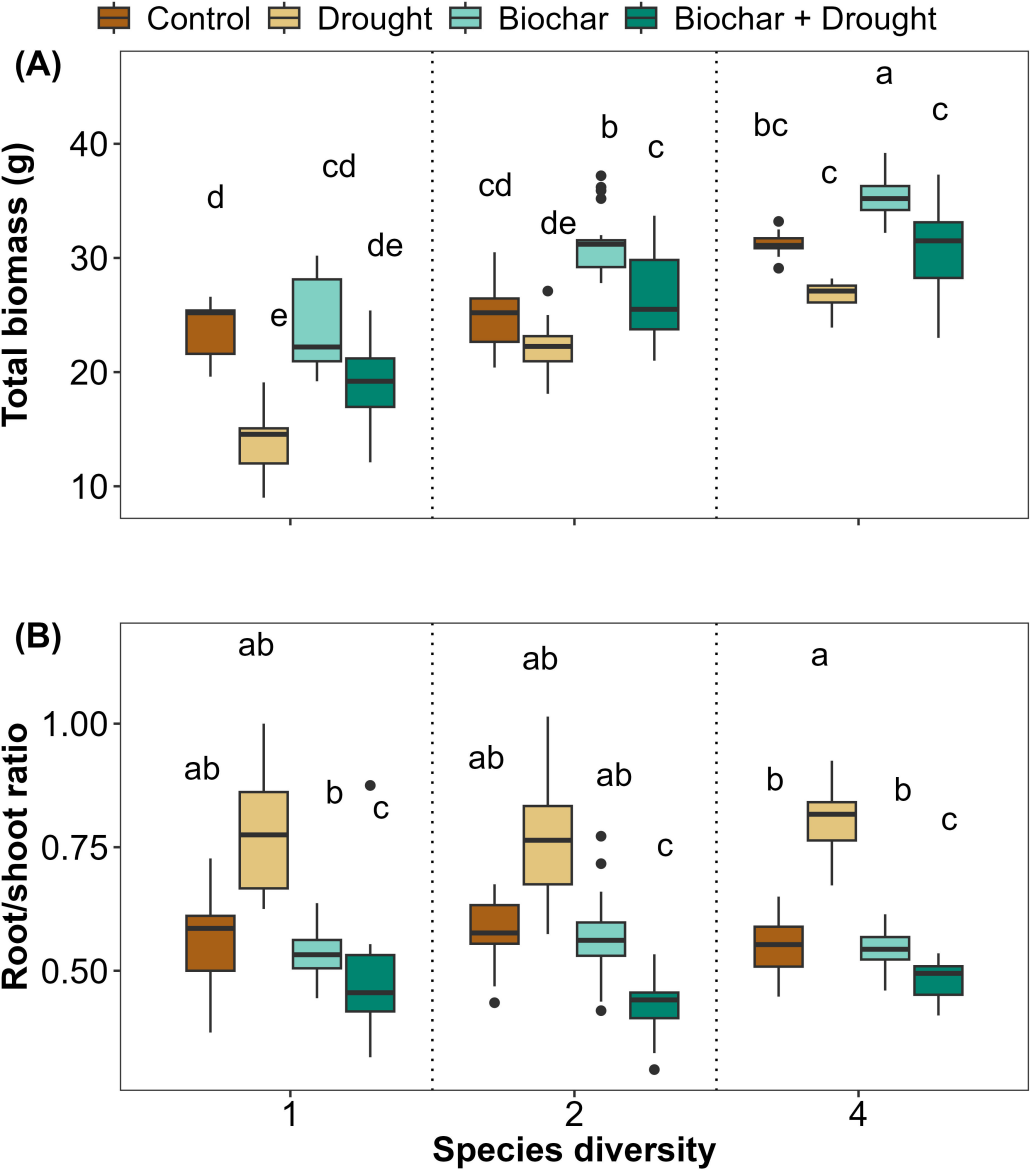
Effects of diversity and biochar application on **(A)** total biomass and **(B)** root/shoot ratio (RSR) under control and drought conditions. Letters indicate significant differences among treatments based on Tukey’s multiple comparison test.

The current results revealed that plants that grew under biochar treatment had higher total biomass than the control; furthermore, drought significantly reduced the total biomass, but this reduced biomass improved in response to biochar even under drought treatment. Likewise, total biomass increased with increasing species diversity (**Figure 3A**; **Supplementary Table S3** in the **Supplementary Materials**). The LMM results showed that 78% of the variance was explained by total biomass, and there was a significant interaction between species diversity, biochar, and drought on their effects on total biomass (**Supplementary Table S3**). The RSR showed the opposite trend to the total biomass, as species that grew under drought conditions had a higher RSR than the other treatments at the three species diversities (**Figure 3B**; **Supplementary Table S3** in the **Supplementary Materials**), with 64% of the variance explained by LMMs for RSR (**Supplementary Table S3**). Drought significantly affected RSR compared to the control, but no other treatments or interactions significantly affected it (**Figure 3B**; **Supplementary Table S3**).

### 3.3 Effects of species diversity, biochar and drought on soil properties

**Figure 4:**
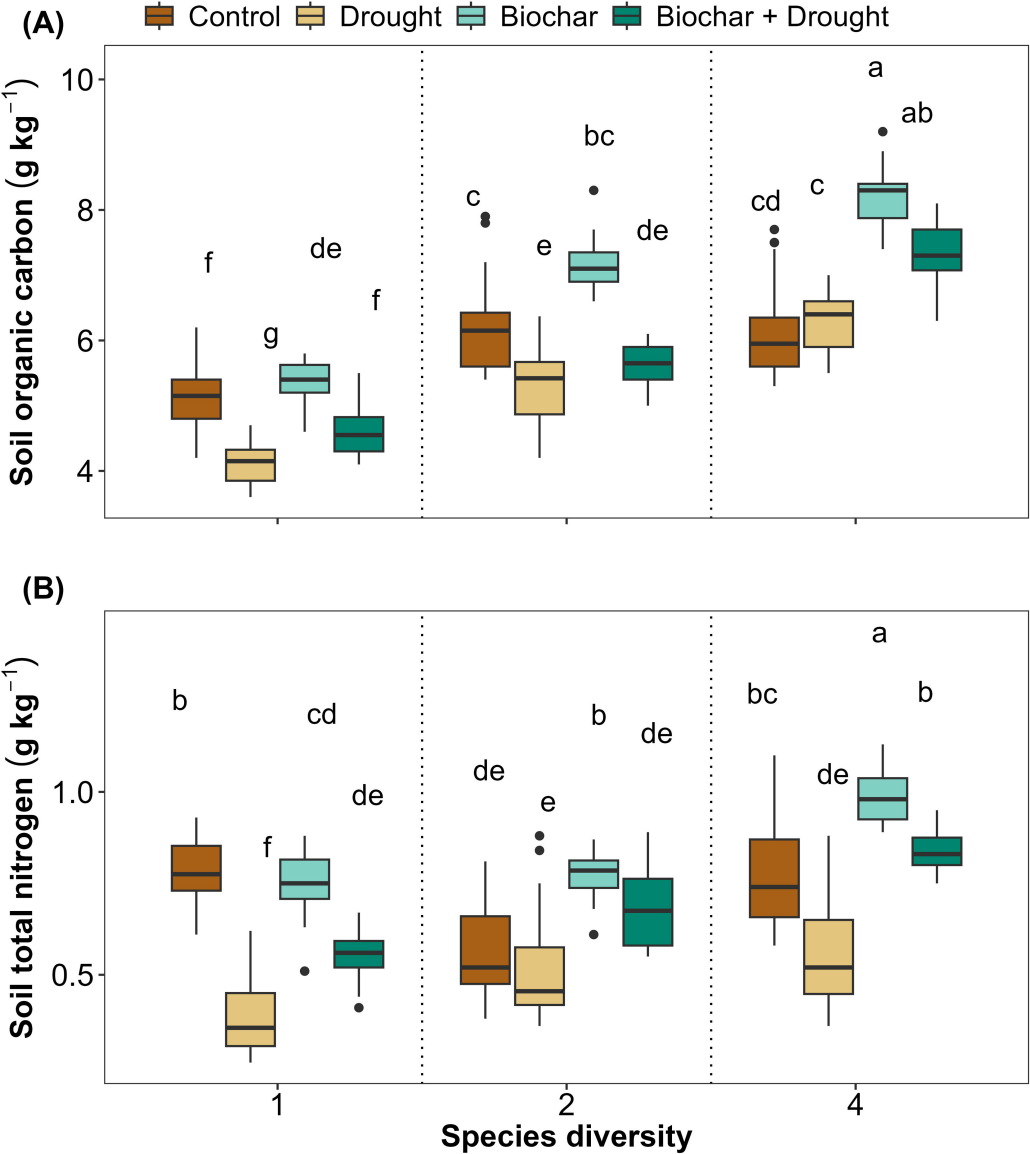
Effects of diversity and biochar application on **(A)** soil organic carbon (SOC) and **(B)** soil total nitrogen (STN) under control and drought conditions. Letters indicate significant differences among treatments based on Tukey’s multiple comparison test.

Diversity, biochar, and drought had significant effects on soil properties (SOC and STN) (**Figures 4A, B**; **Supplementary Table S3** in **Supplementary Materials**). The LMMs explained 84% and 69% of the variance in SOC and STN, respectively. Although diversity and drought significantly affected soil properties, biochar did not show any significant effect on soil properties (**Figures 4A, B**). Finally, the interaction between diversity, biochar, and drought significantly affected STN, but this was not the case for the interaction between 2-species diversity, biochar, and drought on their effects on SOC (**Figures 4A, B**; **Supplementary Table S3** in **Supplementary Materials**).

### 3.4 Effects of species diversity, biochar, and drought on microbial content

**Figure 5:**
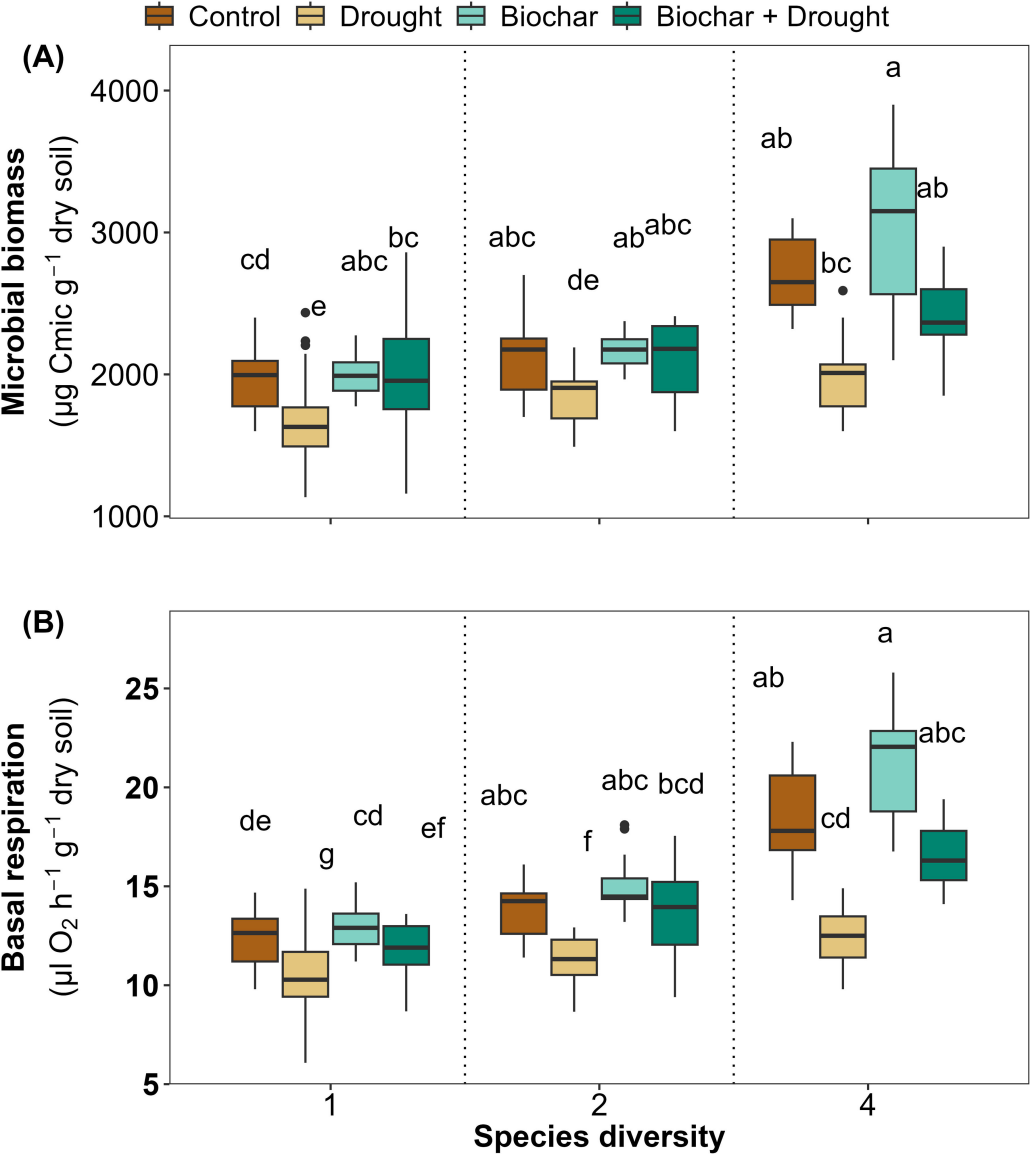
Effects of diversity and biochar application on **(A)** microbial biomass (Cmic) and **(B)** soil basal respiration under control and drought conditions. Letters indicate significant differences among treatments based on Tukey’s multiple comparison test.

The soil microbial biomass showed a significant positive response to the diversity of 4-species, a significant negative response to drought, and a significant negative interaction between 4-species and drought in comparison to the other treatments, and 59% of the variance was explained by LMMs (**Figure 5A**; **Supplementary Table S3** in the **Supplementary Materials**). Soil basal respiration was positively affected by the two species diversity (2 and 4 species) and negatively by drought in comparison to the other treatments, where 73% of the variance was explained by LMMs (**Figure 5B**; **Supplementary Table S3** in the **Supplementary Materials**). Biochar did not significantly affect soil microbial biomass or soil basal respiration, but it improved the soil basal respiration significantly when interacted with the 4-species diversity (**Figures 5A, B**; **Supplementary Table S3** in the **Supplementary Materials**).

### 3.5 Effects of species diversity, biochar and drought on biodiversity effects

**Figure 6:**
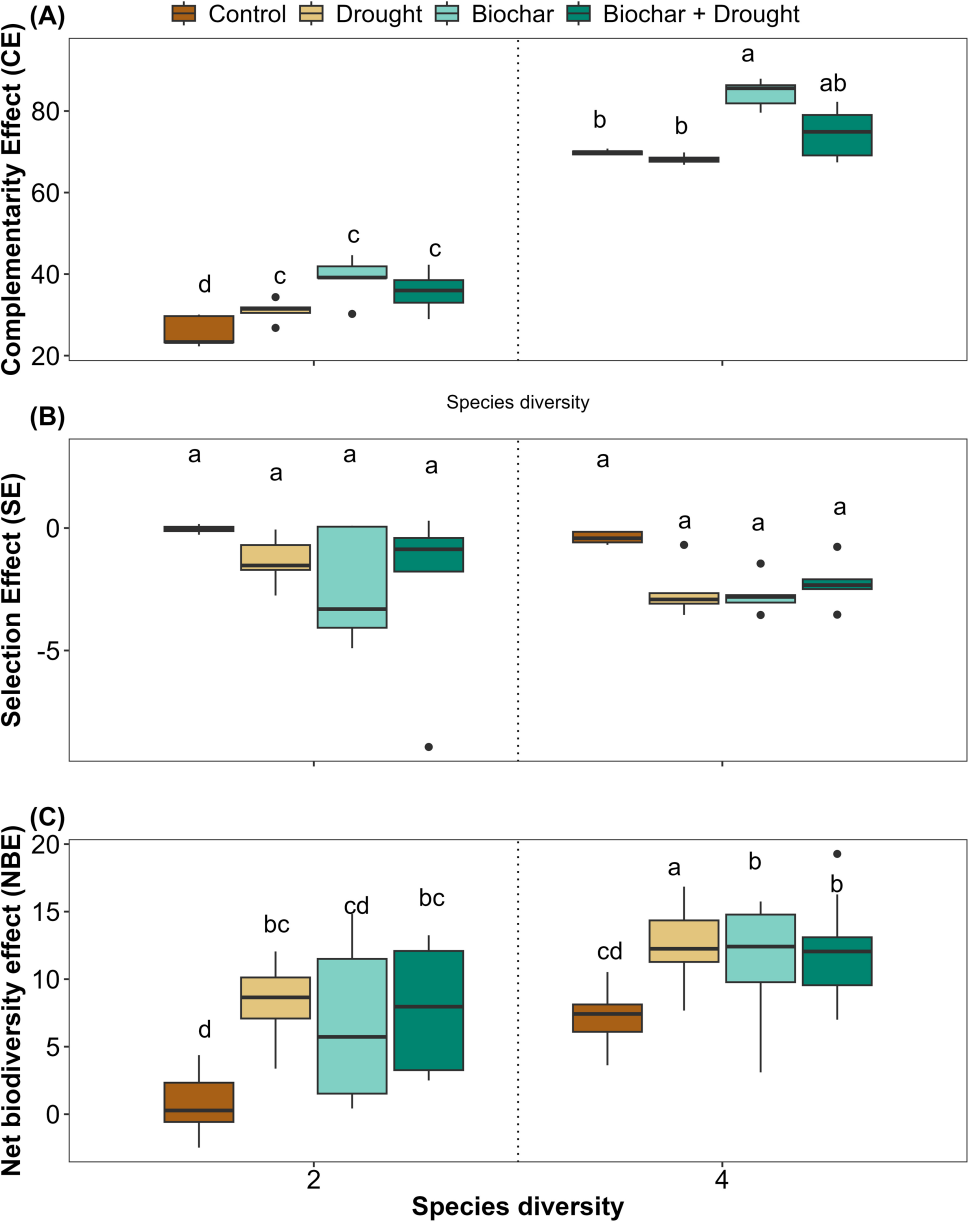
Effects of diversity and biochar application on **(A)** complementarity effect (CE), **(B)** selection effect (SE), and **(C)** net biodiversity effect (NBE) under control and drought conditions. Letters indicate significant differences among treatments based on Tukey’s multiple comparison test.

The complementarity effect values were consistently positive and were significantly affected by species diversity, biochar, and biochar+drought. However, the effects of drought alone and the interactions between diversity, biochar, and drought were not significant compared to the other treatments, with 96% of the variance explained by the LMMs (**Figure 6A**; **Supplementary Table S3** in the **Supplementary Materials**). In contrast, there were no significant effects of any of the treatments on the selection effect; their values were always negative, and only 23% of the variance was explained by the LMMs for the selection effect (**Figure 6B**; **Supplementary Table S3** in the **Supplementary Materials**).

In contrast, the net biodiversity effect was significantly affected by species diversity, drought, biochar, and biochar+drought when compared to the control; however, there were no significant effects of the different interactions on the net biodiversity effect (**Figure 6C**; **Supplementary Table S3** in the **Supplementary Materials**), and 51% of the variance was explained by LMMs for the net biodiversity effect (**Supplementary Table S3**).

## 4 Discussion

The current study is one of the few to explore the combined effects of species diversity and biochar in mitigating drought effects in arid environments by measuring species performance, productivity, soil properties, microbial content, and biodiversity effects. It has been showed that both species diversity and biochar as soil amendments can mitigate drought effects under greenhouse conditions by enhancing species performance (higher plant height, SLA, and SRL), species productivity (high total biomass), soil properties (high SOC and STN), soil microbial content (higher Cmic and basal respiration), and biodiversity effects (complementarity and net biodiversity) of the plant communities.

### 4.1 Effect of species diversity and biochar amendment on species performance and productivity under drought

The present study found that high species diversity buffered the adverse impacts of drought on both species productivity (biomass) and performance (plant functional traits), especially when biochar was added to the soil, confirming that in diverse communities, drought-sensitive species are less affected by dry conditions compared to their performance in monocultures (Wright et al., 2021), which suggest that biodiversity can mitigate harsh environmental conditions and can potentially be used as a tool to protect plant species against several environmental changes such as drought (Chen et al., 2022; Dietrich et al., 2022; Li et al., 2023; Liu et al., 2024; Wang et al., 2024). Biochar amendment has also demonstrated positive effects on plant productivity and performance under drought conditions, as it can improve the biochemical and physiological traits, root architecture, and cellular homeostasis of plants (Bisht et al., 2024).

Regarding biomass production, species produced more biomass as species diversity increased, even under drought conditions, confirming the previous findings of Kreyling et al. (2017) who found that while drought reduced aboveground biomass by 30%, species richness promoted recovery, particularly in low-productivity communities. In another study by van Rooijen et al. (2015) higher plant diversity showed a lower negative response to drought in terms of biomass production. These findings demonstrate that biodiversity can increase ecosystem resistance to drought, with the negative effects being less pronounced in high-diversity plant communities (Wagg et al., 2017; Chen et al., 2022). The results also showed improved biomass in response to biochar amendment, even under drought conditions, confirming the role of biochar in enhancing nutrient supply rather than increasing water uptake by improving soil microbial communities (Hardy et al., 2019). Specifically, biochar application increased nitrogen acquisition through biological nitrogen fixation and enhanced potassium uptake, which may have increased stress tolerance (Mannan et al., 2021).

RSR, which is a measure of plant productivity, was only significantly affected by drought. The highest RSR was obtained under drought and 4-species diversity, suggesting that under drought conditions, species tend to reduce their aboveground biomass rather than reducing their root biomass to adapt to water deficiency and absorb as much water as possible (Lemoine et al., 2013). These findings are consistent with previous studies suggesting that increased species richness enhances multifunctionality and stability of ecosystem functions under drought conditions (Grossiord et al., 2014; Grange et al., 2024).

Plant height, SLA, and SRL, which are considered good measures to detect the effects of diversity and climate change on an ecosystem scale, a significant effect of species diversity on these parameters was detected, as plant height and SLA increased in response to species diversity under both drought and biochar treatments. These results suggest that in communities with high species diversity, species can better utilize light and nutrients owing to the greater variation in plant height, SLA, and SRL (Freschet et al., 2021; Hou et al., 2024). As previously discussed, drought can affect the allocation strategies of plants owing to its effect on soil nutrient availability as a result of water shortage (Knapp et al., 2017), which can be mitigated by improving plant species diversity (Hou et al., 2024). Biochar also improved plant height and SLA under drought conditions, especially in species-rich communities, confirming previous findings that biochar application improves plant performance under drought conditions (Ali et al., 2017b) by increasing the water-holding capacity and physical and biological properties of soils, which can indirectly affect leaf and root characteristics and consequently improve nutrient acquisition (Moles et al., 2009). The current results showed also positive effects of 4-species diversity and its interaction with drought on SRL, which suggest that under high species diversity and drought plants adopt a drought tolerant strategy to allow efficient water absorption capacity (Bristiel et al., 2019), such responses of SRL to drought were not obvious under low species diversity as plants under drought treatment showed lower SRL in comparison to the other treatments, which suggest previous findings that drought reduce SRL in species poor communities (Larson and Funk, 2016; Lozano et al., 2020).

### 4.2 Role of soil properties and microbial content in mitigating drought

In the current study, species diversity and biochar application had significant effects on SOC and STN under drought conditions, which can be discussed as species diversity enhancing SOC and STN storage through increased community productivity and plant-soil feedback mechanisms (Liu et al., 2019), which is important in water-limited environments, as diverse plant communities can better utilize limited soil moisture (Dietrich et al., 2022; Li et al., 2023). Likewise, biochar amendments consistently increase SOC and STN across different soil types and environmental conditions (Han et al., 2016; Dong et al., 2022; Zhang et al., 2022) because the recalcitrant nature of biochar makes it highly stable in soil, contributing directly to the carbon and nitrogen pools in soil (Kimetu and Lehmann, 2010). Under drought conditions, biochar can help retain soil moisture and nutrients by improving soil structure and providing habitat for microorganisms (Wang et al., 2016; Li et al., 2020).

The soil microbial content, species diversity, and biochar application significantly improved Cmic and basal respiration in soil, particularly under drought conditions, which is consistent with several previous studies that showed that higher plant species diversity generally increases microbial content even under drought conditions (Li et al., 2022; Xi et al., 2023; Liu et al., 2024), suggesting that high species diversity can reduce the drought effects by improving soil bacterial and fungal diversity, soil glomalin, and soil enzymes which consequently will increase plant access to nutrients and will improve soil microbial community resistance to several environmental stressors such as drought (Bennett et al., 2020; Li et al., 2022), as soil with a high microbial content can contain taxa that adapt to drought conditions (Wardle et al., 2004).

Biochar amendment also tends to increase Cmic and basal respiration across different ecosystems (Paetsch et al., 2018; Ge et al., 2019). Under drought conditions, biochar amendment can be particularly beneficial as it can enhance microbial biomass and soil enzyme activities under limited water availability (Jabborova et al., 2022).

### 4.3 Net biodiversity, complementarity, and selection effects under different species diversity, biochar amendment, and drought

The current study also demonstrated how species diversity and biochar amendment under drought conditions affect the overall community productivity through NBE, CE, and SE. The results showed significantly higher NBE and CE under the 4-species diversity mixture, which has been observed in several previous studies (Wang et al., 2020a; Hou et al., 2024). Such an increase in CE under high diversity, even under drought, confirms that diversity can mitigate the negative impacts of drought on ecosystem productivity (Fichtner et al., 2020; Chen et al., 2022). Additionally, the current results showed positive NBE and CE and negative SE, which was previously discussed that the increase in biomass is associated with positive CE and negative SE (Craven et al., 2016). Positive values of NBE indicate that species in the 4-species diversity perform better than expected based on their biomass in the 1-speceis diversity, suggesting complementarity or facilitation effect (Chen et al., 2025). Based on these results, CE can be considered a good measure for understanding how biodiversity influences ecosystem functions and reflects species interactions. Moreover, there were no differences in SE, confirming that the current experimental plots did not have any dominant or high-performing species. These results confirm that strong complementarity is more resilient to climate change and can maintain ecosystem functions under drought conditions (Hou et al., 2024; Chen et al., 2025).

## 5 Conclusions

The current study clearly demonstrated that higher species diversity can mitigate the negative effects of drought, especially when the soil is amended with biochar. These effects were evident as high diversity and biochar produced more productive species (higher total biomass and lower RSR), better species performance (taller plants, higher SLA, and SRL), more fertile soil (higher SOC and STN), better microbial content (higher Cmic and basal respiration), and higher net biodiversity and complementarity effects under drought conditions than monoculture and control. These results provide a deeper understanding of drought-biodiversity interactions, which is crucial for predicting how changes in functional composition affect ecosystem functions and the restoration of arid environments in Oman. One of the limitations of the current study is that it was performed under greenhouse conditions, necessitating future studies that may focus on the long-term effects of diversity and different types of biochar under field conditions.

## Data Availability

The original contributions presented in the study are included in the article/**Supplementary Material**. Further inquiries can be directed to the corresponding author/s.
