# Supplementary material for: High species diversity and biochar can mitigate drought effects in arid environments
Source: Front Plant Sci. 2025 May 20;16:1563585. doi: 10.3389/fpls.2025.1563585 (PMC12130053; doi:10.3389/fpls.2025.1563585)
Supplement: Supplementary file 1 [file Table1.docx]

Supplementary Material

**Table S1.** Plant species pool used in the study testing the effect of diversity and biochar application under drought and control conditions.

| **No.** | **Scientific Name** | **Life form** | **Family** |
| --- | --- | --- | --- |
| 1 | *Ranunculus muricatus* | Annual | Ranunculaceae |
| 2 | *Ipomoea obscura* | Perennial | Convolvulaceae |
| 3 | *Pulicaria glutinosa* | Perennial | Asteraceae |
| 4 | *Dicoma schimperi* | Annual | Asteraceae |
| 5 | *Paronychia arabica* | Annual | Caryophyllaceae |

**Table S2.** Species combinations of experimental plant communities involved in the study testing the effect of diversity and biochar application under drought and control conditions.

| Species combinations | | Plant #1 | Plant #2 | Plant #3 | Plant #4 |
| --- | --- | --- | --- | --- | --- |
| Monoculture  (1 species) | S_1 | *Ranunculus muricatus* | *Ranunculus muricatus* | *Ranunculus muricatus* | *Ranunculus muricatus* |
|  | S_2 | *Ipomoea obscura* | *Ipomoea obscura* | *Ipomoea obscura* | *Ipomoea obscura* |
|  | S_3 | *Pulicaria glutinosa* | *Pulicaria glutinosa* | *Pulicaria glutinosa* | *Pulicaria glutinosa* |
|  | S_4 | *Dicoma schimperi* | *Dicoma schimperi* | *Dicoma schimperi* | *Dicoma schimperi* |
|  | S_5 | *Paronychia arabica* | *Paronychia arabica* | *Paronychia arabica* | *Paronychia arabica* |
| Mixture  (2 species) | M_2_1 | *Ranunculus muricatus* | *Ranunculus muricatus* | *Ipomoea obscura* | *Ipomoea obscura* |
|  | M_2_2 | *Ranunculus muricatus* | *Ranunculus muricatus* | *Paronychia arabica* | *Paronychia arabica* |
|  | M_2_3 | *Ipomoea obscura* | *Ipomoea obscura* | *Dicoma schimperi* | *Dicoma schimperi* |
|  | M_2_4 | *Ranunculus muricatus* | *Ranunculus muricatus* | *Pulicaria glutinosa* | *Pulicaria glutinosa* |
|  | M_2_5 | *Pulicaria glutinosa* | *Pulicaria glutinosa* | *Paronychia arabica* | *Paronychia arabica* |
| Mixture  (4 species) | M_4_1 | *Ranunculus muricatus* | *Ipomoea obscura* | *Pulicaria glutinosa* | *Paronychia arabica* |
|  | M_4_2 | *Ipomoea obscura* | *Pulicaria glutinosa* | *Dicoma schimperi* | *Paronychia arabica* |
|  | M_4_3 | *Ranunculus muricatus* | *Ipomoea obscura* | *Dicoma schimperi* | *Paronychia arabica* |
|  | M_4_4 | *Ranunculus muricatus* | *Ipomoea obscura* | *Pulicaria glutinosa* | *Dicoma schimperi* |
|  | M_4_5 | *Ranunculus muricatus* | *Pulicaria glutinosa* | *Dicoma schimperi* | *Paronychia arabica* |

**Table S3.** Estimates, *t*-statistics, *P*-values and *R^2^* for linear mixed effect models testing the effect of diversity and biochar application and the their combinations on total biomass, root-shoot ratio (RSR), soil organic carbon (SOC), soil total nitrogen (STN), soil microbial content (Cmic), soil basal respiration, plant height, specific leaf area (SLA), specific root length (SRL), complementarity effect (CE), selection effect (SE), and net biodiversity effect (NBE) under control and drought conditions. Statistically signiﬁcant variables are indicated in bold.

| **Response variable** | **Explanatory variables** | **Estimates** | ***t*-value** | ***P*** | R^2^ |
| --- | --- | --- | --- | --- | --- |
| **Biomass** | Intercept | 23.94 | 24.65 | **<0.001** | 0.78 |
|  | Diversity (2 species) | 0.96 | 0.70 | 0.485 |  |
|  | Diversity (4 species) | 7.23 | 5.27 | **<0.001** |  |
|  | Drought | -10.10 | -7.35 | **<0.001** |  |
|  | Biochar | 0.02 | 0.01 | 0.988 |  |
|  | Biochar + Drought | -5.23 | -3.80 | **<0.001** |  |
|  | Diversity (2 species) × Drought | 7.28 | 3.75 | **<0.001** |  |
|  | Diversity (4 species) × Drought | 5.61 | 2.89 | **0.004** |  |
|  | Diversity (2 species) × Biochar | 6.34 | 3.26 | **<0.001** |  |
|  | Diversity (4 species) × Biochar | 4.20 | 2.16 | **0.032** |  |
|  | Diversity (2 species) × Biochar + Drought | 7.08 | 3.65 | **<0.001** |  |
|  | Diversity (4 species) × Biochar + Drought | 4.58 | 2.36 | **0.019** |  |
| **RSR** | Intercept | 0.56 | 22.53 | **<0.001** | 0.64 |
|  | Diversity (2 species) | 0.02 | 0.54 | 0.587 |  |
|  | Diversity (4 species) | -0.01 | -0.32 | 0.750 |  |
|  | Drought | 0.22 | 6.22 | **<0.001** |  |
|  | Biochar | -0.02 | -0.62 | 0.536 |  |
|  | Biochar + Drought | -0.07 | -1.85 | 0.066 |  |
|  | Diversity (2 species) × Drought | -0.03 | -0.53 | 0.595 |  |
|  | Diversity (4 species) × Drought | 0.04 | 0.76 | 0.448 |  |
|  | Diversity (2 species) × Biochar | 0.01 | 0.27 | 0.786 |  |
|  | Diversity (4 species) × Biochar | 0.02 | 0.36 | 0.722 |  |
|  | Diversity (2 species) × Biochar + Drought | -0.08 | -1.69 | 0.093 |  |
|  | Diversity (4 species) × Biochar + Drought | 0.00 | -0.02 | 0.980 |  |
| **SOC** | Intercept | 5.10 | 42.01 | **<0.001** | 0.84 |
|  | Diversity (2 species) | 1.09 | 6.32 | **<0.001** |  |
|  | Diversity (4 species) | 1.07 | 6.23 | **<0.001** |  |
|  | Drought | -1.01 | -5.88 | **<0.001** |  |
|  | Biochar | 0.26 | 1.49 | 0.139 |  |
|  | Biochar + Drought | -0.46 | -2.68 | **0.008** |  |
|  | Diversity (2 species) × Drought | 0.13 | 0.54 | 0.590 |  |
|  | Diversity (4 species) × Drought | 1.16 | 4.76 | **<0.001** |  |
|  | Diversity (2 species) × Biochar | 0.76 | 3.13 | **0.002** |  |
|  | Diversity (4 species) × Biochar | 1.81 | 7.45 | **<0.001** |  |
|  | Diversity (2 species) × Biochar + Drought | -0.10 | -0.41 | 0.681 |  |
|  | Diversity (4 species) × Biochar + Drought | 1.63 | 6.71 | **<0.001** |  |
| **STN** | Intercept | 0.79 | 31.60 | **<0.001** | 0.69 |
|  | Diversity (2 species) | -0.22 | -6.19 | **<0.001** |  |
|  | Diversity (4 species) | -0.02 | -0.68 | 0.496 |  |
|  | Drought | -0.40 | -11.22 | **<0.001** |  |
|  | Biochar | -0.04 | -1.02 | 0.308 |  |
|  | Biochar + Drought | -0.24 | -6.76 | **<0.001** |  |
|  | Diversity (2 species) × Drought | 0.35 | 7.12 | **<0.001** |  |
|  | Diversity (4 species) × Drought | 0.18 | 3.60 | **<0.001** |  |
|  | Diversity (2 species) × Biochar | 0.24 | 4.75 | **<0.001** |  |
|  | Diversity (4 species) × Biochar | 0.26 | 5.25 | **<0.001** |  |
|  | Diversity (2 species) × Biochar + Drought | 0.35 | 6.99 | **<0.001** |  |
|  | Diversity (4 species) × Biochar + Drought | 0.32 | 6.39 | **<0.001** |  |
| **Cmic** | Intercept | 1956.00 | 22.59 | **<0.001** | 0.59 |
|  | Diversity (2 species) | 145.50 | 1.19 | 0.236 |  |
|  | Diversity (4 species) | 761.00 | 6.21 | **<0.001** |  |
|  | Drought | -277.50 | -2.27 | **0.024** |  |
|  | Biochar | 57.50 | 0.47 | 0.639 |  |
|  | Biochar + Drought | 55.00 | 0.45 | 0.654 |  |
|  | Diversity (2 species) × Drought | -6.50 | -0.04 | 0.970 |  |
|  | Diversity (4 species) × Drought | -467.50 | -2.70 | **0.007** |  |
|  | Diversity (2 species) × Biochar | 15.00 | 0.09 | 0.931 |  |
|  | Diversity (4 species) × Biochar | 281.00 | 1.62 | 0.106 |  |
|  | Diversity (2 species) × Biochar + Drought | -74.00 | -0.43 | 0.670 |  |
|  | Diversity (4 species) × Biochar + Drought | -376.00 | -2.17 | **0.031** |  |
| **Basal respiration** | Intercept | 12.30 | 26.85 | **<0.001** | 0.73 |
|  | Diversity (2 species) | 1.53 | 2.36 | **0.019** |  |
|  | Diversity (4 species) | 6.21 | 9.58 | **<0.001** |  |
|  | Drought | -1.92 | -2.96 | **0.003** |  |
|  | Biochar | 0.71 | 1.10 | 0.273 |  |
|  | Biochar + Drought | -0.55 | -0.84 | 0.401 |  |
|  | Diversity (2 species) × Drought | -0.71 | -0.78 | 0.437 |  |
|  | Diversity (4 species) × Drought | -4.21 | -4.60 | **<0.001** |  |
|  | Diversity (2 species) × Biochar | 0.40 | 0.44 | 0.661 |  |
|  | Diversity (4 species) × Biochar | 2.15 | 2.35 | **0.020** |  |
|  | Diversity (2 species) × Biochar + Drought | 0.35 | 0.38 | 0.703 |  |
|  | Diversity (4 species) × Biochar + Drought | -1.46 | -1.59 | 0.112 |  |
| **Plant height** | Intercept | 61.10 | 32.85 | **<0.001** | 0.76 |
|  | Diversity (2 species) | 13.60 | 5.17 | **<0.001** |  |
|  | Diversity (4 species) | 20.70 | 7.87 | **<0.001** |  |
|  | Drought | -8.15 | -3.10 | **0.002** |  |
|  | Biochar | 28.80 | 10.95 | **<0.001** |  |
|  | Biochar + Drought | 2.55 | 0.97 | 0.333 |  |
|  | Diversity (2 species) × Drought | -2.65 | -0.71 | 0.477 |  |
|  | Diversity (4 species) × Drought | 2.75 | 0.74 | 0.460 |  |
|  | Diversity (2 species) × Biochar | -4.45 | -1.20 | 0.233 |  |
|  | Diversity (4 species) × Biochar | -5.55 | -1.49 | 0.137 |  |
|  | Diversity (2 species) × Biochar + Drought | -7.20 | -1.94 | 0.054 |  |
|  | Diversity (4 species) × Biochar + Drought | -3.00 | -0.81 | 0.421 |  |
| **SLA** | Intercept | 2.59 | 27.66 | **<0.001** | 0.86 |
|  | Diversity (2 species) | 1.14 | 8.62 | **<0.001** |  |
|  | Diversity (4 species) | 1.57 | 11.82 | **<0.001** |  |
|  | Drought | -1.00 | -7.52 | **<0.001** |  |
|  | Biochar | 1.18 | 8.88 | **<0.001** |  |
|  | Biochar + Drought | 0.38 | 2.89 | **<0.001** |  |
|  | Diversity (2 species) × Drought | 0.24 | 1.27 | 0.206 |  |
|  | Diversity (4 species) × Drought | 0.00 | -0.01 | 0.989 |  |
|  | Diversity (2 species) × Biochar | 0.03 | 0.17 | 0.275 |  |
|  | Diversity (4 species) × Biochar | 0.04 | 0.20 | 0.831 |  |
|  | Diversity (2 species) × Biochar + Drought | 0.25 | 1.32 | 0.958 |  |
|  | Diversity (4 species) × Biochar + Drought | 0.10 | 0.55 | 0.576 |  |
| **SRL** | Intercept | 23.71 | 52.00 | **<0.001** | 0.71 |
|  | Diversity (2 species) | 0.36 | 0.55 | 0.584 |  |
|  | Diversity (4 species) | 2.24 | 3.47 | **0.001** |  |
|  | Drought | -7.08 | -10.97 | **<0.001** |  |
|  | Biochar | 0.12 | 0.18 | 0.859 |  |
|  | Biochar + Drought | -3.90 | -6.05 | **<0.001** |  |
|  | Diversity (2 species) × Drought | 2.50 | 2.74 | **0.009** |  |
|  | Diversity (4 species) × Drought | 5.61 | 6.15 | **<0.001** |  |
|  | Diversity (2 species) × Biochar | 0.75 | 0.82 | 0.418 |  |
|  | Diversity (4 species) × Biochar | 2.84 | 3.11 | **0.003** |  |
|  | Diversity (2 species) × Biochar + Drought | 1.23 | 1.34 | 0.185 |  |
|  | Diversity (4 species) × Biochar + Drought | 3.67 | 4.03 | **<0.001** |  |
| **CE** | Intercept | 25.51 | 14.20 | **<0.001** | 0.96 |
|  | Diversity (4 species) | 44.38 | 17.39 | **<0.001** |  |
|  | Drought | 5.06 | 1.99 | 0.055 |  |
|  | Biochar | 13.05 | 5.14 | **<0.001** |  |
|  | Biochar + Drought | 9.79 | 3.85 | **0.001** |  |
|  | Diversity (4 species) × Drought | -6.80 | -1.88 | 0.069 |  |
|  | Diversity (4 species) × Biochar | 1.33 | 0.37 | 0.715 |  |
|  | Diversity (4 species) × Biochar + Drought | -5.13 | -1.42 | 0.165 |  |
| **SE** | Intercept | 0.00 | 0.00 | 0.997 | 0.23 |
|  | Diversity (4 species) | -0.40 | -0.43 | 0.667 |  |
|  | Drought | -0.65 | -1.32 | 0.196 |  |
|  | Biochar | -0.73 | -1.49 | 0.147 |  |
|  | Biochar + Drought | -0.56 | -1.14 | 0.264 |  |
|  | Diversity (4 species) × Drought | -1.53 | -1.18 | 0.246 |  |
|  | Diversity (4 species) × Biochar | -1.59 | -1.23 | 0.229 |  |
|  | Diversity (4 species) × Biochar + Drought | -1.29 | -0.99 | 0.328 |  |
| **NBE** | Intercept | 0.81 | 0.77 | 0.44 | 0.51 |
|  | Diversity (4 species) | 6.43 | 4.99 | **<0.001** |  |
|  | Drought | 7.43 | 5.00 | **<0.001** |  |
|  | Biochar | 5.73 | 3.85 | **0.001** |  |
|  | Biochar + Drought | 7.01 | 4.72 | **<0.001** |  |
|  | Diversity (4 species) × Drought | -1.82 | -1.00 | 0.32 |  |
|  | Diversity (4 species) × Biochar | -1.53 | -0.84 | 0.41 |  |
|  | Diversity (4 species) × Biochar + Drought | -2.44 | -1.34 | 0.19 |  |
